# Si-Based Anode Materials for Li-Ion Batteries: A Mini Review

**DOI:** 10.1007/s40820-014-0008-2

**Published:** 2014-09-27

**Authors:** Delong Ma, Zhanyi Cao, Anming Hu

**Affiliations:** 1 Institute of Laser Engineering, Beijing University of Technology, 100 Pingle Yuan, Chaoyang District, Beijing, 100124 People’s Republic of China; 2 Key Laboratory of Automobile Materials, Ministry of Education and School of Materials Science and Engineering, Jilin University, Changchun, 130012 People’s Republic of China; 3 Department of Mechanical, Aerospace and Biomedical Engineering, University of Tennessee, 1512 Middle Drive, Knoxville, TN 37996-2210 USA

**Keywords:** Li-ion batteries, Anode, Si, High capacity, Nanomaterials

## Abstract

Si has been considered as one of the most attractive anode materials for Li-ion batteries (LIBs) because of its high gravimetric and volumetric capacity. Importantly, it is also abundant, cheap, and environmentally benign. In this review, we summarized the recent progress in developments of Si anode materials. First, the electrochemical reaction and failure are outlined, and then, we summarized various methods for improving the battery performance, including those of nanostructuring, alloying, forming hierarchic structures, and using suitable binders. We hope that this review can be of benefit to more intensive investigation of Si-based anode materials.

## Introduction

In the last two decades, the Li-ion batteries (LIBs) have successfully captured the portable electronic market. However, when it is proposed to conquer the upcoming markets of electric vehicles, storage of energy from renewable energy sources, such as photovoltaic plants and/or wind turbines and other KWh levels load, great improvements in storage capacity, which is currently mainly limited by their electrode materials, are urgently needed [1–5]. It is well known that the commercial graphite anode cannot meet these challenges due to its low theoretical capacity (372 mAh g^−1^). There is a consensus that the breakthrough in capacity can be achieved by moving from classical intercalation reaction to alloying reaction because the alloying reaction can store more Li compared with intercalation reaction. For example, Li can react with Si to form Li_22_Si_5_ alloy, but with graphite only, to form LiC_6_ alloy. Since Dey demonstrated that Li metal can electrochemically alloy with other metals (Sn, Pb, Al, Au, Pt, Zn, Ag, Mg, and Cd) at room temperature [6], Li-alloying reactions with metallic or semi-metallic elements and various compounds have been investigated during the past few decades, such as Sn, P, Ge, Pb, and Sb. Wen et al. showed that Sn reacted with lithium to yield different Li–Sn phases: Li_2_Sn_5_, LiSn, Li_7_Sn_3_, Li_5_Sn_2_, Li_13_Sn_5_, Li_7_Sn_2_, and Li_22_Sn_5_. A black P/C nanocomposite also showed high capacity (about 2,000 mAh g^−1^) [3]. Among the various Li alloy elements, Si has been considered as one of the most attractive anode materials for LIBs, not only because of its high gravimetric (4,200 mAh g^−1^) and volumetric capacity (2,400 mAh cm^−3^), but also due to its abundance, cheapness, and environmentally benign property, as shown in Table 1. However, it suffers from fast capacity fading, which greatly hampers the application of Si anode materials.

**Table 1:** Property of Li alloy elements [3]

Element	Gravimetric capacity (mAh g^−1^)	Volumetric capacity (mAh cm^−3^)	Cost	Toxicity	Safety
Si	4,200	2,400	Low	No	High
C	372	890	Low	No	Low
Ge	1,568	2,300	High	High	High
Sn	990	2,020	Low	No	High
P	2,600	2,250	Low	High	Low
Sb	660	1,890	Low	High	Low
Pb	549	1,790	Low	High	Low

### The Mechanism of Electrochemical Lithiation

LIBs are mainly composed of anode (generally graphite), a carbonate-based organic electrolyte, and a cathode (generally LiCoO_2_). Li ions are intercalated and deintercalated between graphite and LiCoO_2_ through the electrolyte during discharge and charge. The theoretical capacities of anode and cathode are 372 mAh g^−1^ (graphite) and less than 160 mAh g^−1^ (LiCoO_2_), respectively, which are too low, especially for anode material. Si anode is very attractive because of its high theoretical capacity of 4,200 mAh g^−1^ which is 10 times more than that of commercial graphite [3]. Moreover, the discharging potential is about 0.2 V with respect to Li/Li^+^, which is lower than most of other alloy-type and metal oxide anodes [7]. Furthermore, it is safer and stabler than graphite (lithiated silicon is more stable in typical electrolytes than lithiated graphite) [8].

**Fig. 1:**
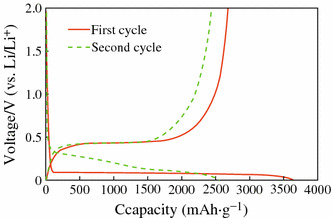
Voltage profiles of Si powder electrode for the first and second discharge/charge cycles [18]

The mechanism of electrochemical lithiation of Si is critical to improve the performance of Si anode, which has been investigated by several groups [9–16]. It is found that the reactions follow the equilibrium Li–Si binary phase diagram at high temperature, forming different intermetallic compounds and showing distinct voltage plateaus for each two-phase region [17]. However, there is only a two-phase region at about 0.1 V at room temperature during first discharge process [18], as shown in Fig. 1. It should be noted that the two-phase region disappears after first cycle. In order to find out the lithiation mechanism, X-ray diffraction (XRD) analysis was performed to investigate the phase transition [13–15], and the reaction mechanism is explained as follows:

During discharge

1 $$\text{Si}\;\left(\text{crystalline}\right)\;+\;x{\text{Li}}^{+}\;+\;x{\text{e}}^{-}\to \;{\text{Li}}_{x}\text{Si}\;\text{(amorphous)}\;+\;\left(3.75-x\right)\;{\text{Li}}^{+}\;+\;\left(3.75-x\right)\;{\text{e}}^{-}$$

2 $$\to {\text{Li}}_{15}{\text{Si}}_{4}(\text{crystalline)}$$

During charge

3 $${\text{Li}}_{15}{\text{Si}}_{4}(\text{crystalline)}\to \;\text{Si(amorphous) +}\;y{\text{Li}}^{+}+y{\text{e}}^{-}+{\text{Li}}_{15}{\text{Si}}_{4}(\text{residual)}$$

In the two-phase region, crystalline Si becomes amorphous Li–Si alloy during the first lithiation (1), and the highly lithiated amorphous Li_*x*_Si phase is suddenly found to crystallize into Li_15_Si_4_ phase around 60 mV (vs. Li/Li^+^) (2). Another two-phase region appears during the first delithiation process, and the final product is amorphous Si (3). There are also amounts of residual Li_15_Si_4_ phase after the first delithiation, which can be avoided if the potential of the Si electrode is controlled above 70 mV during cycling. When Li ions react with the amorphous Si during the second cycle, the two-phase region disappears, and sloping voltage plateaus are observed, which indicates single-phase region. After the second cycle, reactions (2) and (3) were repeated, show the above features repeatedly, and reversible capacity faded quickly.

### The Failure Mechanism

**Fig. 2:**
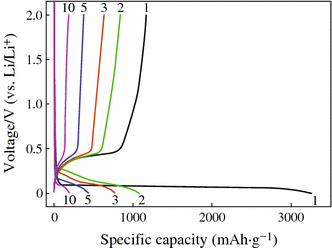
Charge–discharge voltage profiles of Si powder anode [16]

**Fig. 3:**
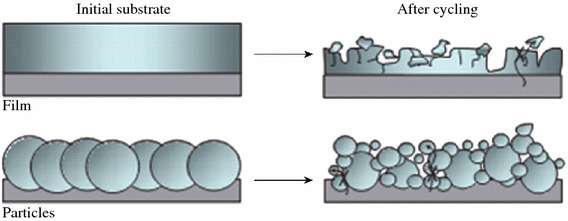
Schematic of morphologic changes that occur in Si during electrochemical cycling [20]
- Solid electrolyte interphase (SEI) stability at the interface between the silicon and the liquid electrolyte is also responsible for the failure of the Si anode. The SEI layer is formed during battery discharging, due to electrolyte decomposition on the surface of anode at the low potential. As shown in Fig. 4, a thin layer of SEI is formed in the lithiated and expanded state [21]. During delithiation, the Si particle shrinks, and the SEI layer breaks down into separate pieces, and fresh Si surface is exposed to the electrolyte. In later cycles, new SEI layer continues to be formed on the newly exposed silicon surfaces. The SEI is an electronic insulator but a Li–ion conductor, and so the growth of the SEI layer is eventually terminated at a certain thickness. The thick SEI layer is harmful for the cycle life, because it can cause a rise of the electrode impedance/polarization and decrease of the electrode’s electrochemical reactivity. As discussed above, a large volume change and unstable formation of SEI film are the main issues for the failure of Si anode.

Although Si has the highest theoretical capacity, its cycling performance is very poor. Figure 2 shows the charge–discharge profiles of Si powder anode at a current density of 100 mA g^−1^. It could be found that a large amount of irreversible capacity appears in the first cycle. The first discharging capacity is about 3,260 mAh g^−1^ but that of the charging is only 1,170 mAh g^−1^. After 10 cycles, only very low capacity (about 200 mAh g^−1^) can be retained. To understand the reasons for the poor cycling stability of Si anode, the failure mechanism has been investigated by several groups [16, 19]. The conclusions can be drawn as follows:

- The large change in the volume of Si anodes, which increases internal resistance and loss of contact area between Si and conductive materials, is considered to be the main reason for their rapid capacity loss. Figure 3 shows the schematic of morphologic changes that occur in Si during electrochemical cycling [20]. The volume of Si anodes changes by about 400 % during cycling. As a result, Si films and particles tend to undergo pulverization during cycling. Most of the material loses contact with the current collector, resulting in poor transport of electrons.

**Fig. 4:**
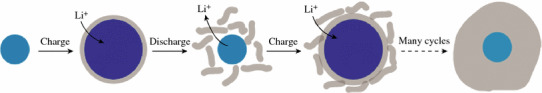
Schematic of SEI formation on silicon surfaces [21]

## The Methods to Improve the Battery Performance

### Si Nanostructures

**Fig. 5:**
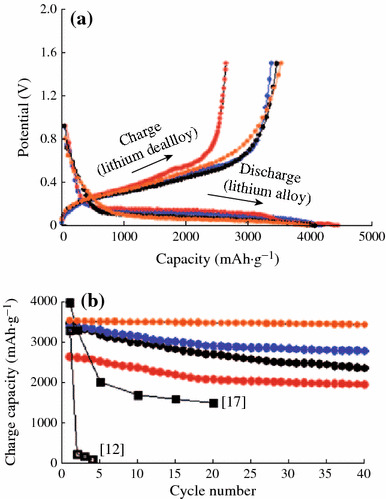
**a** Voltage profiles of 5, 10, and 20-nm-sized Si and 10-nm-sized Si after carbon coating during the first cycle in coin-type half-cells at a rate of 0.2 C between 0 and 1.5 V. **b** Plot of charge capacity versus cycle number (*red*: 5 nm, *blue*: 10 nm, *orange*: 10 nm after carbon coating, *black circles*: 20 nm) [24]. (Color figure online)

Tremendous efforts have been made to improve the batteries performance of Si anode. In order to overcome the volume change during electrochemical reaction, many researches are focused on accommodating the volume changes in the earlier studies. Nanomaterials have the genuine potential to make a significant impact on the electrochemical performance of Si anode [22], as their reduced dimensions enable far higher intercalation/deintercalation rates. In addition, the volume change can be also buffered after downsizing the Si particle to nano-size. The significance of nano-sized Si on battery performance was demonstrated by several groups. Li et al. reported that a nano-Si (78 nm) powder anode showed better capacity retention than bulk Si powder [23]. Kim et al. also synthesized Si nanoparticles with various sizes (5, 10, and 20 nm) and studied their battery performance [24]. The results indicated that 10-nm-sized Si showed the highest capacity retention among the samples, as shown in Fig. 5.

**Fig. 6:**
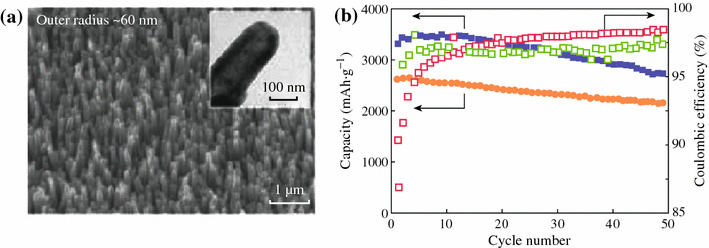
**a** SEM and TEM *images* (*insets*) of the sealed tip of a Si nanotubes. **b** Cycle performances at a rate of 0.05 and 0.2 C (*square*: 0.05 C, *circle*: 0.2 C) [25]

One-dimensional (1D) nanowires and nanotubes are also intriguing structures with good cycle stability. Cui et al. synthesized Si nanowires which were grown directly on the metallic current collector substrate [20]. The limited nanowire diameter allows for better accommodation of the large volume change and provides 1D electronic pathways allowing for efficient charge transport. The Si nanowires display high capacities at higher current density. Even at the 1 C rate, the capacities remain 2,100 mAh g^−1^, and a reversible capacity of over 3,000 mAh g^−1^ is maintained after 10 cycles. An array of sealed Si nanotubes is also prepared by CVD of Si on to ZnO nanorods and selective removal of ZnO (see Fig. 6) [25]. It shows discharge capacities of 3,360 and 2,500 mAh g^−1^ at the rates of 0.05 and 0.2 C respectively, and high capacity retentions (about 81 and 82 % at 0.05 and 0.2 C, respectively) after 50 cycles.

Two-dimensional (2D) Si thin film is another promising nanostructure with improved cycle stability and rate capabilities, which can minimize the volume variation and retain structural integrity [26–29]. Of course, the battery performance depends on the film thickness as thinner films deliver larger accommodation capacity. For example, a 50-nm-thick Si film is found to deliver a higher discharge capacity and better cycling performance compared with 150-nm-thick Si film [26]. Although Si thin film offers high specific capacity, good capacity retention and fast charge/discharge rate, the practical application is hampered because of their prohibitively high synthesis costs and low active material content.

**Fig. 7:**
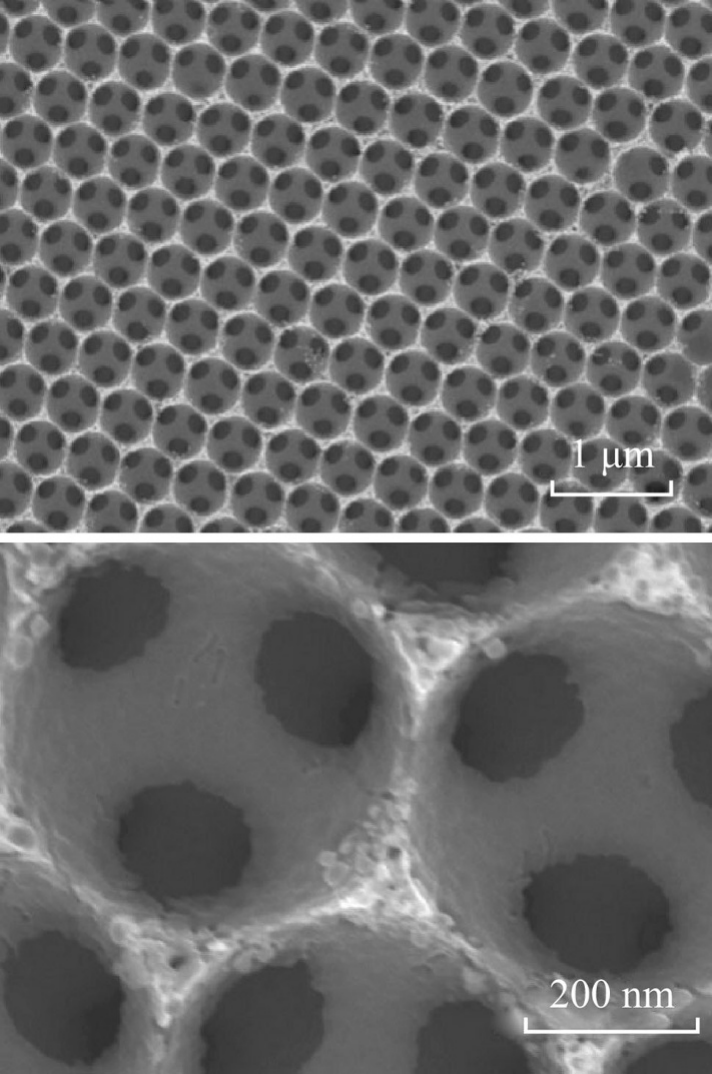
SEM *images* of the nanocrystalline Si inverse opal structure [36]

Recently, three-dimensionally macroporous (3DM) structure materials used in LIBs have attracted more attention, due to their special nature [30–35], as shown in Fig. 7. First, the wall thicknesses are on the order of nanometers to tens of nanometers, which can shorten both electronic and ionic pathways. Second, macropores with a size range of several hundred nanometers enable easy infiltration of electrolyte and fast liquid-phase Li ion diffusion, reducing the concentration polarization and increasing rate performance and capacity of the cell. Third, the continuous network of electrode material may provide better electrical conductivity than aggregates of loosely connected particles. Finally, macroporosity should help in accommodating volume change during cycling without losing the structural integrity of the electrode. Esmanski et al. synthesized several types of silicon-based inverse-opal films and studied their electrochemical performances [36]. These electrodes demonstrated significant improvement both in capacity retentions and rate capabilities.

**Fig. 8:**
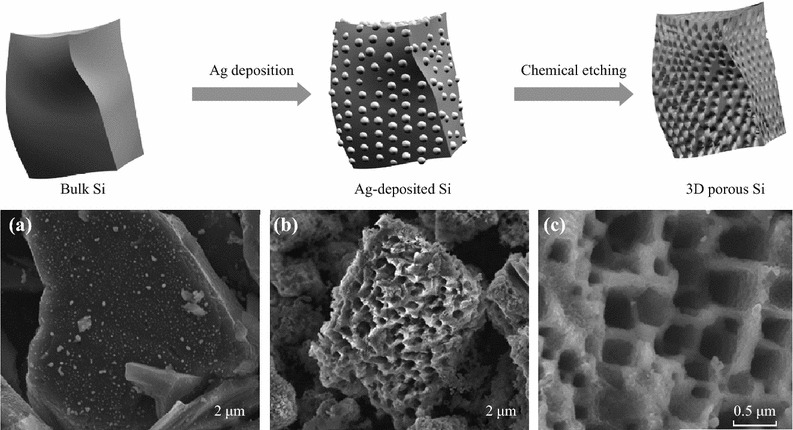
Upper: schematic illustration of the preparation of macroporous Si powders. Ag nanoparticles were deposited onto the surface of bulk silicon via a galvanic reaction, and subsequently, the Ag-deposited Si was chemically etched to make 3D porous Si particles. Lower: **a** SEM *image* of Ag-deposited Si. **b** SEM *image* of chemically etched Si. **c** Magnified SEM *image* of samples seen in (**b**) [37]

**Fig. 9:**
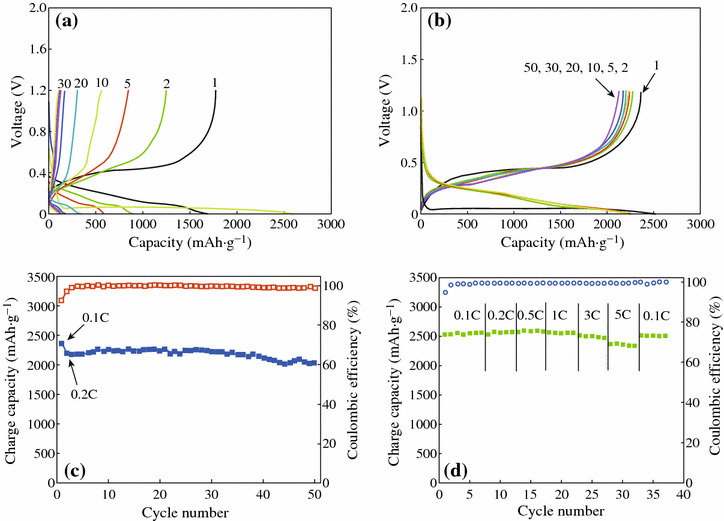
Electrochemical evaluation of macroporous Si and the carbon-coated macroporous Si. **a** Voltage profiles of chemically etched Si anodes at 0.1 C rate between 0.005 and 1.2 V in coin-type half cell. **b** Voltage profiles of the carbon-coated porous Si anodes at 0.1 C (the first cycle) and 0.2 C (2nd–50th cycles) in the same conditions. **c** Plot of charge capacity versus cycle number obtained from voltage profiles seen in (**b**). **d** Rate capabilities of the carbon-coated porous Si anodes [37]

Bang et al. reported a simple route for synthesizing 3DM bulk Si materials by combining an electroless metal deposition via a galvanic displacement reaction, with a metal-assisted chemical etching process using commercially available bulk Si powders (Fig. 8). The as-prepared materials exhibited a high reversible capacity of approximately 2,050 mAh g^−1^ with a remarkable initial coulombic efficiency of 94.4 %, and stable cycling retention (Fig. 9) [37].

### Si/M Composites (M: Active/inactive Conductive Materials)

In comparison with bulk Si materials, the pristine nanostructures of Si have shown improved capacity retention. However, the nano-sized Si anode materials are still plagued with the intrinsic low electrical conductivity of Si. Liu et al. have shown that increasing the conducting additive content played an important role in improving battery performance of the electrodes [38].

 Several groups have paid attention to some intermetallic compounds containing Si as anode materials, such as Mg_2_Si, CaSi_2_, NiSi, FeSi, CoSi_2_, FeSi_2_, and NiSi_2_ [12, 39–44]. For example, the first discharge capacity of NiSi is shown to be 1,180 mAh g^−1^, which corresponds to the insertion of 3.82 mol Li, and the initial coulombic efficiency was 80 %. Here, Ni is expected to act as a buffering and conductive matrix for the formation of Li_*x*_Si in the subsequent cycles. Nano-Si/polypyrrole (PPy) composites are prepared by HEMM and chemical polymerization, respectively [45]. It is found that the PPy conducting polymer matrix is effectively acting to increase the electrical conductivity and buffer the volume change.

**Fig. 10:**
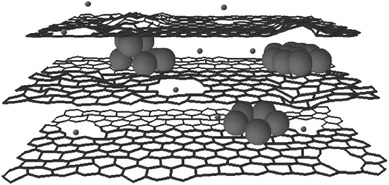
The schematic drawing of a section of a composite electrode material constructed with a graphitic scaffold with in-plane carbon vacancy defects [51]

**Fig. 11:**
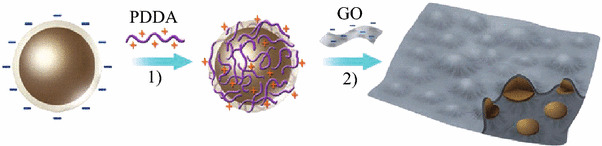
Schematic process for fabricating the Si/graphene nanocomposite [52]

Recently, some works on Si–C composites have been reported. When graphite and pyrolyzed carbon are used as matrices, some promising results have been obtained in terms of initial coulombic efficiency, high reversible capacity, and cycling stability. Carbon is expected to play an important role in electrode reliability owing to its relative softness, small volume change during Li insertion, and good electronic conductivity. Graphene is a good candidate to host active nanoparticles among the various carbon materials because of its high surface area, superior electrical conductivity, and excellent mechanical flexibility [46–49]. In general, graphene sheets in the as-obtained hybrid materials can function as supports of active materials and provide conductive channels for electrons through the electrodes. Chou et al. prepared Si/graphene composite by sample mixing of nano-sized Si and graphene [50]. The improved cycling stability is attributed to the good mechanical properties and conductivity provided by graphene. Zhao et al. reported Si/graphene composite constructed with a graphitic scaffold with in-plane carbon vacancy defects [51]. Figure 10 shows the schematic drawing. They demonstrated the beneficial effects of nanometer-sized in-plane vacancies on ion transport for use in a 3D graphitic scaffold that can be fabricated into hybrid materials with a combination of power capability and storage capacity for battery electrode applications. Zhou et al. used a self-assembly approach to encapsulate Si nanoparticles in graphene via electrostatic force (Fig. 11) [52]. A good cycling performance of the composite had been observed. The capacity reached 1,205 mAh g^−1^ after 150 cycles.

### Hollow and Yolk–Shell Structure Composites

Pioneering works have shown that decreasing the feature size to the nanoscale allows for the material to withstand the large (de) lithiation strains without fracture. However, the cycle life of nano-sized silicon is still limited due to the unstable SEI on the surface. Recently, hollow and yolk–shell structures of Si composites, coated with conductive materials, have been shown to be an efficient way to solve the problem [53–58].

**Fig. 12:**
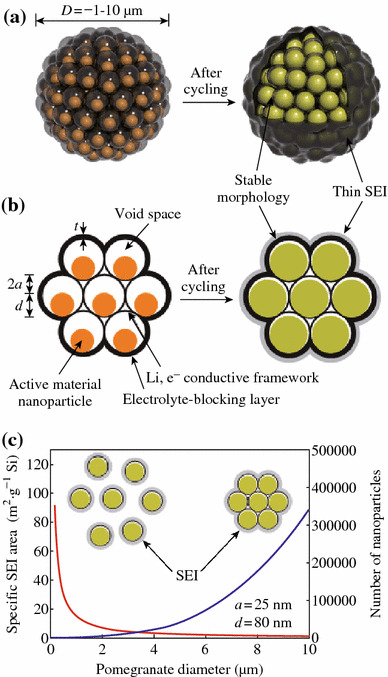
Schematic of the pomegranate-inspired design. 3D view (**a**) and simplified 2D cross-sectional view (**b**) of one pomegranate microparticle before and after electrochemical cycling (in the lithiated state). **c** Calculated surface area in contact with electrolyte (specific SEI area) and the number of primary nanoparticles in one pomegranate particle versus its diameter [59]

**Fig. 13:**
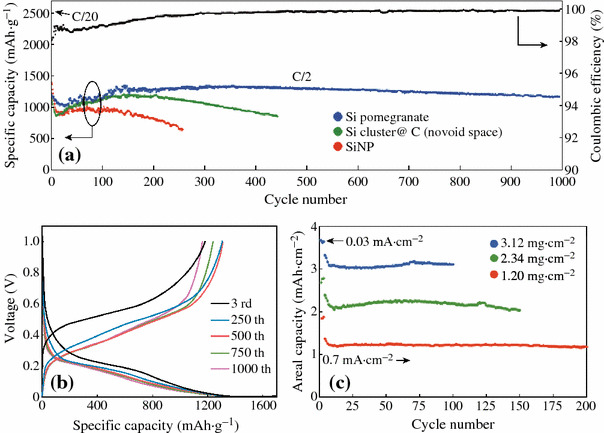
Electrochemical characterization of Si pomegranate anodes. **a** Reversible delithiation capacity for the first 1,000 galvanostatic cycles of the Si pomegranate and other structures tested under the same conditions. Coulombic efficiency is plotted for the silicon pomegranate only. The rate was C/20 for the first cycle and C/2 for later cycles. **b** Voltage profiles for the Si pomegranate plotted for the 3rd, 250th, 500th, 750th, and 1,000th cycles. **c** High areal mass loading test (up to 3.12 mg cm^−2^ active material) of silicon pomegranate anodes [59]

Cui et al. demonstrated a novel secondary structure for Si anodes [59], as shown in Fig. 12. Such a design has multiple advantages: (1) the nano-sized primary particle and the well-defined internal void space allow the silicon to expand; (2) the carbon framework functions as an electrical highway so that all nanoparticles are electrochemically active; (3) carbon completely encapsulates the entire secondary particle, limiting the SEI film formation to the outer surface, which not only limits the amount of SEI, but also retains the internal void space for silicon expansion; and (4) the dilemmas of high surface area and low tap density, which are introduced when using nano-sized primary features, are partially solved. As shown in Fig. 13a, its reversible capacity reaches 2,350 mAh g^−1^ at a rate of C/20, and after 1,000 cycles, over 1,160 mAh g^−1^ capacity can be reached. The average coulombic efficiency from the 500th to 1000th cycles of the Si pomegranate is as high as 99.87 %, indicating that SEI is very stable.

**Fig. 14:**
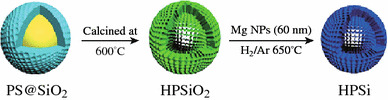
Scheme of preparing hollow porous Si nanoparticles [60]

Chen et al. reported a facile approach to fabricate mono-disperse hollow porous Si (HPSi) nanoparticles (ca. 120 nm) by the magnesiothermic reduction of hollow porous SiO_2_ (HPSiO_2_) nanoparticles [60]. This step was followed by Ag coating the nanoparticle for conductivity enhancement (Fig. 14). The HPSi anode showed a high specific reversible capacity (3,762 mAh g^−1^), good cycle stability (over 93 % capacity retention after 99 cycles), and high rate performance (over 2,000 mAh g^−1^ at a current density 4,000 mA g^−1^).

### Effect of Binder

Recent studies have also shown that many important battery characteristics, including stability and irreversible capacity losses, are critically dependent on the binder’s properties [61–64]. It is found that polymers containing carboxy groups, such as polyacrylic acid (PAA) and carboxymethyl cellulose (CMC), demonstrate promising characteristics as binders for Si-based anodes [65, 66]. The polar hydrogen bonds between the carboxy groups of the binder and the SiO_2_ on the Si surface are proposed to exhibit a self-healing effect and reform if locally broken.

**Fig. 15:**
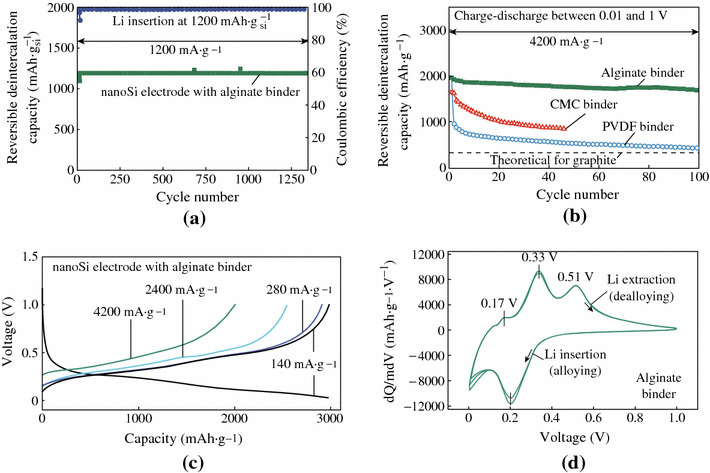
Electrochemical performance of alginate-based nano-Si electrodes. **a** Reversible Li-extraction capacity and CE of the nano-Si electrodes versus cycle number for Li insertion level fixed to 1,200 mAh g^−1^ Si. **b** Reversible Li-extraction capacity of nano-Si electrodes with alginate, CMC, and P VDF binders versus cycle number collected for the current density of 4,200 mA g^−1^ for cells cycled in the potential window of 0.01 - 1 V versus Li/Li^+^. **c** Galvanostatic discharge profiles of the nano-Si electrode at different current densities between 0 and 1 V. **d** Differential capacity *curves* of the nano-Si electrode in the potential window of 0 - 1 V versus Li/Li^+^ collected at the rate of 0.025 mV s^−1^ after the first galvanostatic charge–discharge cycle [67]

Kovalenko et al. used alginate as the binder of nano-Si electrode, which yielded a remarkably stable battery anode [67]. As shown in Fig. 15, charge–discharge cycling performance with Li insertion capacity limited to 1,200 mAh g^−1^ shows stable anode performance for more than 1,300 cycles. At a current density of 4,200 mA g^−1^, the reversible Li extraction specific capacity of an alginate-based Si anode ranges from 1,700 to 2,000 mAh g^−1^ (Fig. 15b). At a smaller current density of 140 mA g^−1^ (Fig. 3c), the specific capacity of the Si anode reaches 3,040 mAh g^−1^. The good battery performance can be contributed to several reasons. First, a very weak binder–electrolyte interaction prevents access of the solvent molecules to the Si–binder boundary. Second, the alginate can provide access of Li ions to the Si surface because it is an ion conductor. Third, it also can assist in building a deformable and stable SEI on the Si surface.

## Summary and Outlook

To sum up, we have briefly reviewed the recent advances in the studies on the Si anode materials. Although Si has an extremely high theoretical capacity, the bulk Si powders have demonstrated very poor electrochemical performance, due to the large volume variation during the charge and discharge processes, resulting in pulverization and delamination from the current collectors. Reducing the materials size to nanoscale is an effective method to solve this problem. For example, diverse nanostructured Si (0D, 1D, 2D, and 3D), Si/metal nanocomposites, and Si/C nanocomposites show improved battery performance. However, these methods cannot form stable SEI on the surface of Si, which is another important reason for the failure of Si anode. Experimental results show that surface modification is a practical method to control the formation of SEI. For example, hollow and yolk–shell structures of Si composites, coated with conductive materials, show excellent battery performance.

Although considerable advances have been achieved in the last decade to design and synthesize Si-based anode materials, for the practical application composite, several fundamental issues remain to be solved. First, nanomaterials have the genuine potential to make a significant impact on the performance of LIBs. However, they are certainly not a panacea. For example, coulombic efficiency of nanopowder is very low, because the high electrolyte/electrode surface area leads to more significant side reactions. Further, the density of nanopowder is generally less than the same material formed from micrometer-sized particles, which would reduce the volumetric energy density of battery. The problem mentioned above should be solved in the subsequent researches. Possible strategies include the following: (1) fabricating hierarchical nano/micrometer-sized structures to improve the tap density of electrode materials and building ultrathick electrodes to enhance the overall volumetric capacity [68–70]; (2) adopting surface coating to avoid unnecessary side reactions [71, 72]; and (3) using advanced manufacturing methods, such as 3D printing, to fabricate electrodes with 3D battery architecture by optimizing the structures of both anodes and cathodes [73, 74].

In addition, the electrolyte offers a promising field for more extensive research efforts. It is found that the electrolyte containing VC or FEC has been recognized to favor the formation of more stable SEI film. Furthermore, the fabrication cost of nano-structured Si remains high and needs to be reduced for its practical applications.
